# Conductive Hydrogels in Biomedical Engineering: Recent Advances and a Comprehensive Review

**DOI:** 10.3390/gels12010069

**Published:** 2026-01-13

**Authors:** Chenyu Shen, Ying Wang, Peng Yuan, Jinhuan Wei, Jingyin Bao, Zhangkang Li

**Affiliations:** Basic Medical Research Center, Medical School, Nantong University, Nantong 226001, China; 2531310002@stmail.ntu.edu.cn (C.S.); 2231510012@stmail.ntu.edu.cn (Y.W.); ntdxmzkyp@ntu.edu.cn (P.Y.); ruthwei@ntu.edu.cn (J.W.)

**Keywords:** hydrogels, biomedical applications, conductivity, biocompatibility, conductive polymers, ionic conductivity, bioelectronics, neural engineering

## Abstract

Conductive hydrogels have gained considerable interest in the biomedical field because they provide a soft, hydrated, and electrically active microenvironment that closely resembles native tissue. Their unique combination of electrical conductivity and biocompatibility enables monitoring and modulation of biological activities. With the rapid development of conductive hydrogel technologies in recent years, a comprehensive overview is needed to clarify their biological functions and the latest biomedical applications. This review first summarizes the fundamental design strategies, fabrication methods, and conductive mechanisms of conductive hydrogels. We then highlight their applications in wearable device, implanted bioelectronics, wound healing, neural regeneration and cell regulation, accompanied by discussions of the underlying biological and electroactive mechanisms. Potential challenges and future directions, including strategies to optimize fabrication methods, balance key material properties, and tailor conductive hydrogels for diverse biomedical applications, are also highlighted. Finally, we discuss the existing limitations and future perspectives of the biomedical applications of conductive hydrogels. We hope that this article may provide some useful insights to support their further development and potential biomedical applications.

## 1. Introduction

The physicochemical characteristics of native soft tissues are closely mimicked by hydrogels, which are three-dimensional networks of polymers rich in water. Their high water content, tunable mechanics, permeability to biomolecules, and intrinsic biocompatibility make them widely used in biomedical engineering [1]. By adjusting polymer composition and crosslinking strategies, hydrogels can be engineered with customizable stiffness, viscoelasticity, degradation rates, adhesiveness, and responsiveness to chemical or physical stimuli [2]. These versatile properties allow hydrogels to create cell-supportive microenvironments resembling the extracellular matrix, enabling applications in wound dressings [3], drug reservoirs [4], tissue scaffolds [5], and soft biointerfaces [6].

To further expand their functionality and adapt hydrogels to increasingly complex biomedical needs, extensive efforts have been devoted to hydrogel functionalization. Through chemical modification, physical blending, or incorporation of functional nanomaterials, hydrogels can be endowed with enhanced mechanical strength [7], self-healing ability [8], adhesiveness [9], injectability [10], photothermal activity [11], and stimuli responsiveness [12]. Functionalized hydrogels also allow dynamic interactions with biological environments, enabling controlled drug release, mechanical signal transduction, immune modulation, and biochemical sensing. These advances have significantly broadened the scope of hydrogel-based technologies and laid the foundation for the development of next-generation soft bioelectronic interfaces. Nevertheless, to achieve efficient electrical coupling with tissues, building upon the advantages of conventional hydrogels, conductive hydrogels have emerged as a specialized subclass designed to bridge electrical communication between biological tissues and engineered devices. By incorporating ionic or electronic conductive pathways, these hydrogels can efficiently transmit, sense, and modulate electrical signals while maintaining tissue-like softness [13]. Ionic conductive hydrogels rely on the mobility of ions (e.g., Na^+^, K^+^, Cl^−^) within the hydrogel network to carry charge, which makes them highly biocompatible and suitable for interfacing with biological tissues. In contrast, electronic conductive hydrogels incorporate conjugated polymers or conductive fillers (e.g., graphene, carbon nanotubes) that allow electron transport, enabling higher conductivity and potential use in electronic devices, sensors, and bioelectronics. Conductive hydrogels overcome the mechanical mismatch between rigid electronic components and dynamic biological systems, creating intimate, stable, and low-impedance interfaces. This unique combination of electrical activity and mechanical compliance enables precise interaction with electrically excitable tissues such as nerves, muscles, and the heart [14].

**Figure 1:**
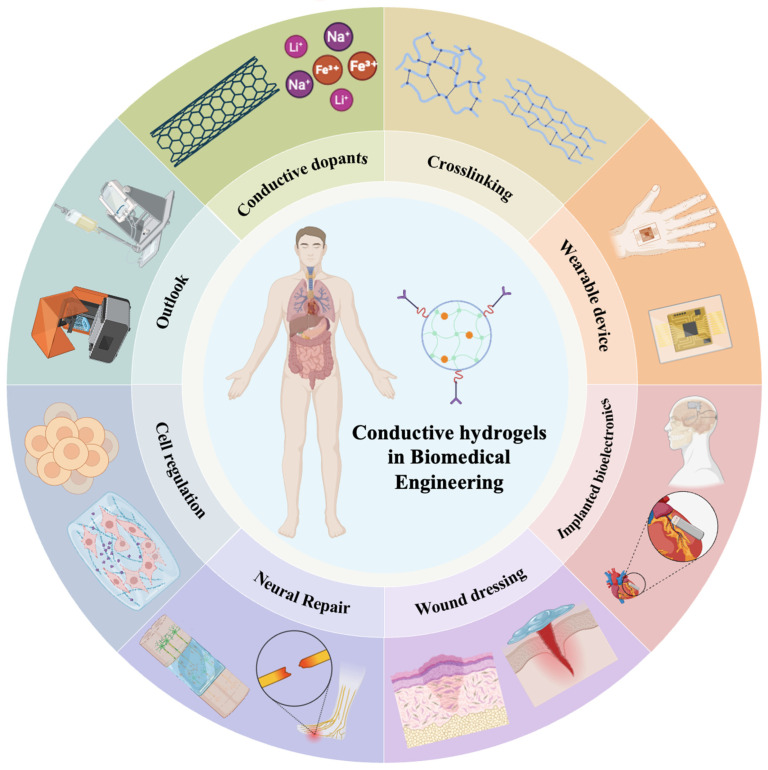
A brief introduction to hydrogels applied in biomedical engineering, enabling applications in wearable devices, implanted bioelectronics, wound dressing, neural repair, cell regulation.

Given the rapid development of this field and its growing biomedical relevance, a comprehensive review is needed to clarify their design principles, functional mechanisms, and emerging applications. We begin by reviewing the key design concepts, fabrication routes, and conductive mechanisms of conductive hydrogels. Their applications in wearable systems, implantable electronics, wound healing, neural repair, and cell regulation are then discussed, with emphasis on the biological and electroactive interactions that enable these functions. We further analyze major challenges, including immunogenicity, long-term biocompatibility, material degradation, and the stability of conductive networks in vivo. Finally, we outline the limitations and future directions that will shape the clinical and translational prospects of conductive hydrogel technologies (Figure 1).

## 2. Fabrication of Conductive Hydrogels

**Figure 2:**
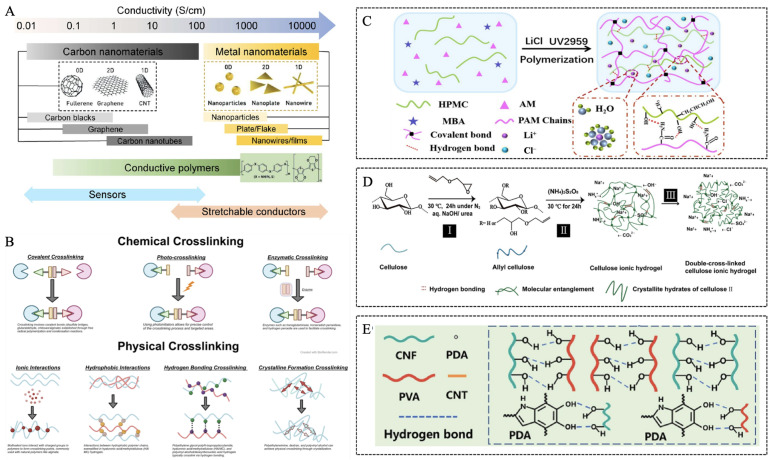
(**A**) Common conductive materials [33] (Adapted with permission from Peng et al. Copyright 2021 American Chemical Society). (**B**) Crosslinking strategies for hydrogels [35] (Copyright © 2024, Gan et al. under the terms of the Creative Commons CC BY 4.0). (**C**) Synthesis of lithium-ion-based conductive hydrogels [36] (Copyright © 2025, Wu et al. under the terms of the Creative Commons CC BY 4.0). (**D**) Fabrication of ionically conductive hydrogels [37] (Adapted with permission from Tong et al. Copyright 2019 American Chemical Society). (**E**) Synthesis of carbon nanotube-doped hydrogels [38] (Adapted with permission from Zhang et al. Copyright 2023 Elsevier).

Substantial interest in nanoparticles and nanomaterials in recent years is due to their unique physicochemical properties and diverse applications in areas such as drug delivery [15,16,17,18,19,20], cancer therapy [21,22,23], cell behavior regulation [24,25,26,27,28], inflammation and bacterial inhibition [29,30], neuroregeneration [31], and food safety monitoring [32]. Beyond these biomedical applications, nanomaterials also contribute significantly to the advancement of conductive soft hydrogels. A common fabrication strategy for conductive hydrogels involves incorporating conductive components into polymer precursor solutions before gelation. Conductive nanofillers such as metal nanoparticles, carbon nanotubes (CNTs), and MXenes enable electron transport through mechanisms including free electron movement, delocalized π-electron systems, and high in-plane electron mobility within layers (Figure 2A) [33]. Despite their advantages, the incorporation of nanoparticles into hydrogels is often accompanied by challenges such as particle agglomeration that compromises uniformity, potential cytotoxic effects arising from surface chemistry or leaching, and limited long-term stability and retention under in vivo physiological conditions. When well-dispersed, these fillers form interconnected percolation networks that convert insulating hydrogels into conductive materials. The point at which percolation begins is influenced by several factors, including the degree of filler dispersion, the aspect ratio of the particles, their tendency to aggregate, and any modifications applied to their surface. In addition to nanofillers, intrinsically conductive polymers such as PEDOT:PSS and polyaniline can be introduced into hydrogel precursor solutions to provide electronic conductivity based on their conjugated molecular structures [34]. Ionic doping presents another effective approach, in which ions such as Li^+^, Na^+^, Fe^3+^, or Al^3+^ migrate through the hydrogel matrix under an electric field and contribute to ionic conductivity. For clearer presentation, the relevant conclusions are summarized in Table 1.

Most conductive additives, regardless of type, are typically blended into the precursor solution before the sol–gel transition. Subsequent crosslinking immobilizes the conductive components within the polymer network, and the process primarily occurs through either chemical or physical crosslinking (Figure 2B) [35]. Chemical crosslinking relies on covalent bond formation through small-molecule crosslinkers, enzymatic reactions, or photo-initiated processes. For photo-initiated reactions, the underlying mechanism also involves covalent crosslinking; carbon–carbon double bonds in the precursor undergo light-induced polymerization with other double bonds to generate crosslinking points. Physical crosslinking, in contrast, arises from noncovalent interactions such as hydrogen bonding, ionic coordination, hydrophobic aggregation, crystallization, or supramolecular association. These strategies enable precise control over conductivity, mechanical behavior, and stability. Recent studies have illustrated the versatility of these approaches. For example, Qin et al. prepared an ionically conductive hydrogel by incorporating LiCl into a polyacrylamide and hydroxypropyl methylcellulose matrix. The resulting material exhibited a conductivity of 9.82 S m^−1^ and demonstrated strong adhesion, high stability, and robust mechanical performance. The introduction of mobile ions enhanced the tensile strain to 1453%, tensile strength to 135 kPa, and elasticity to 9.18 kPa, enabling the development of skin-like sensors with high sensitivity (Figure 2C) [36]. In another report, Tong et al. synthesized double-crosslinked cellulose ionic hydrogels through free radical polymerization of allyl cellulose using ammonium persulfate, combined with NaCl-induced physical crosslinking [37]. These hydrogels maintained nearly 100% tensile strain even at −24 °C and remained visually transparent between −30 and −16 °C. With a conductivity of 1.8 × 10^−5^ S cm^−1^ and excellent temperature tolerance, they offer significant potential for flexible electronics operating under extreme environmental conditions (Figure 2D) [37]. A separate study, inspired by mussel adhesion, developed a conductive composite hydrogel by polymerizing a polyvinyl alcohol system containing polydopamine, cellulose nanofibers, and CNTs (Figure 2E) [38]. Polydopamine enhanced both adhesion and self-healing, while promoting uniform CNTs dispersion through noncovalent interactions. This process produced a conductive, mechanically robust, and antibacterial composite with a conductivity of 0.4 S m^−1^.

## 3. Wearable Device

**Figure 3:**
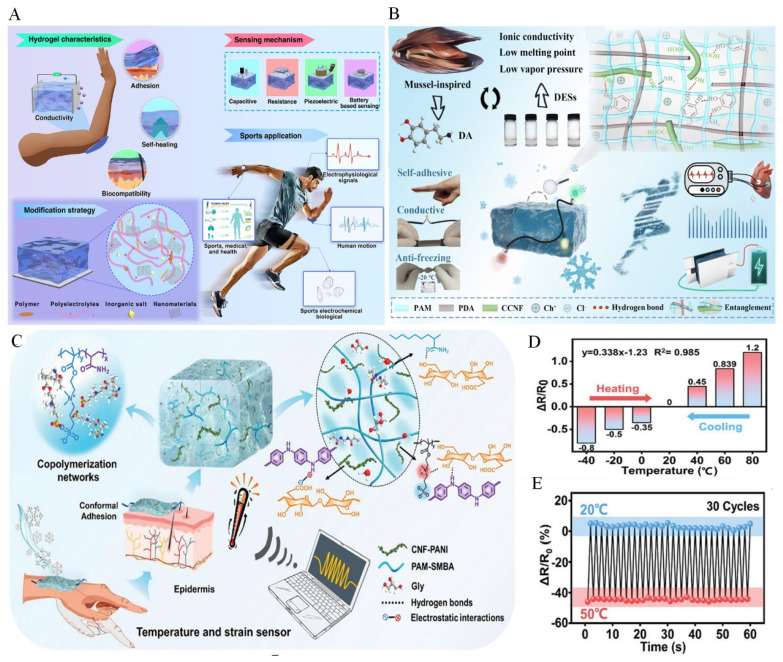
(**A**) Brief introduction to conductive hydrogels for wearable electronic applications [36] (Copyright © 2025, Wu et al. under the terms of the Creative Commons CC BY 4.0). (**B**) Overview of the design strategy, performance and applications of DES/CCNF hydrogels [40] (Copyright © 2024, Li et al. under the terms of the Creative Commons CC BY 4.0). (**C**) Schematic illustration of the PBA/CPA/Gly zwitterionic hydrogel-based temperature sensor [41] (Adapted with permission from Hao et al. Copyright 2022 Elsevier). (**D**) Linear thermal response of the PBA/CPA/Gly hydrogel from −40 to 80 °C [41] (Adapted with permission from Hao et al. Copyright 2022 Elsevier). (**E**) Dynamic temperature response of the PBA/CPA/Gly hydrogel at 20 °C and 50 °C over 30 cycles [41] (Adapted with permission from Hao et al. Copyright 2022 Elsevier).

Conductive hydrogels are most widely used in wearable electronics, particularly for human motion monitoring [39]. The fundamental sensing mechanism is based on mechanical deformation of the hydrogel network induced by human movements such as arm bending, finger flexing, walking, or running. These deformations alter the length, contact area, or arrangement of conductive pathways, resulting in reproducible resistance changes. The corresponding electrical signals can be continuously collected in real time, enabling accurate monitoring of various human motions (Figure 3A) [36].

Beyond high conductivity, conductive hydrogels used in wearable devices should also provide excellent biocompatibility, strong skin adhesion, antifreezing capability, and sufficient mechanical strength to withstand long-term deformation during skin attachment, depending on specific application demands. For example, Li et al. developed a polyacrylamide and polydopamine (PAAm/PDA) hydrogel synthesized in a mixed medium of deep eutectic solvent (DES) and alkaline solution (Figure 3B) [40]. This medium promotes the oxidative polymerization of dopamine, endowing the hydrogel with strong adhesion to biological tissues (20.20 kPa), while retaining the ionic conductivity and low melting point characteristics of DES, enabling stable antifreezing performance at −20 °C. In addition, the incorporation of carboxylated cellulose nanofibers (CCNFs) significantly enhances the mechanical strength through noncovalent interactions, physical entanglement, and interfacial friction, resulting in an approximately threefold improvement. The resulting hydrogel functions effectively as a skin-mounted electrode for electrocardiogram monitoring and as a strain sensor for tracking human motion, even after storage at −20 °C for 15 days. It also demonstrates promising performance as an electrolyte material for supercapacitors. With the increasing functional complexity of conductive hydrogels, strain-induced resistance changes alone are no longer sufficient to meet the demands of next-generation wearable devices. The sensing capabilities of conductive hydrogels are expanding from strain-only detection to multimodal sensing of temperature, humidity, and pressure, enabling more comprehensive physiological and environmental monitoring. For instance, Hao et al. fabricated a zwitterionic PBA/CPA/Gly hydrogel with dual strain and temperature sensing capability through a one-pot copolymerization method (Figure 3C) [41]. The hydrogel exhibits a linear and stable thermal response across a wide temperature range from −40 °C to 80 °C, with consistent thermal sensitivity (Figure 3D). The temperature sensing originates from the temperature-dependent ionic migration and polymer chain mobility within the zwitterionic network. Variations in temperature modulate ion conductivity and chain interactions, producing predictable resistance changes. The hydrogel maintains stable electrical output during 30 cycles of temperature switching between 20 °C and 50 °C, demonstrating its reliable temperature sensing performance (Figure 3E).

## 4. Implanted Bioelectronics

**Figure 4:**
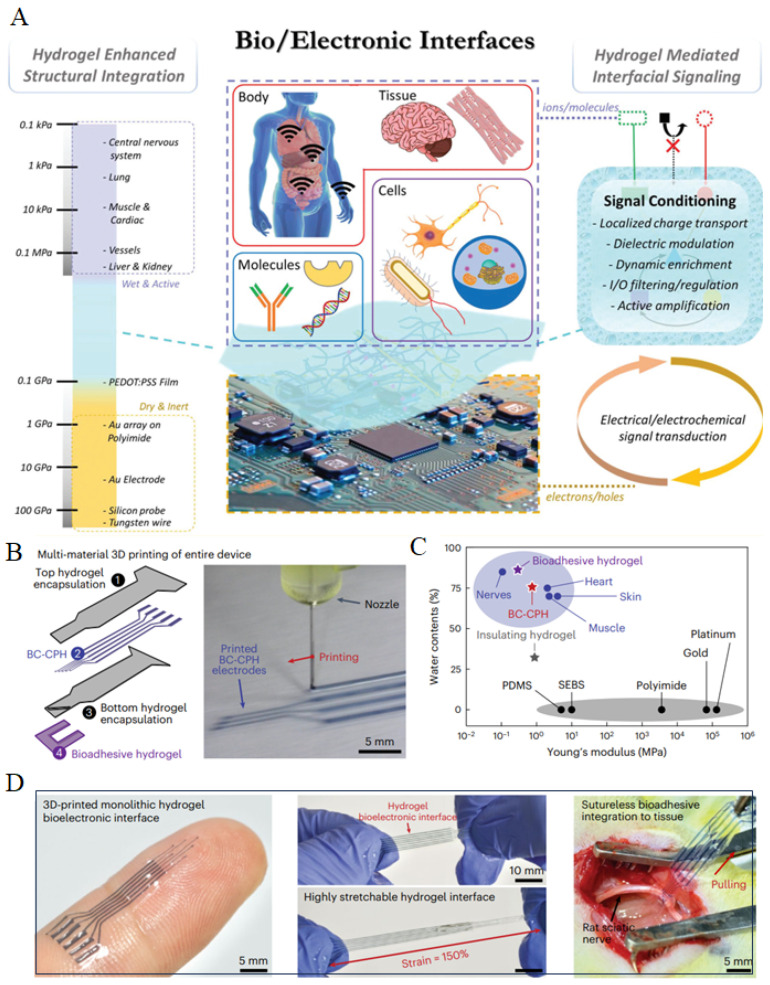
(**A**) Brief introduction to conductive hydrogels for implanted bioelectronics, including their ability to better conform to various organs and to conduct and modulate signal [42] (Copyright © 2021, Richard et al. under the terms of the Creative Commons CC BY 4.0). (**B**) Composition and preparation of conductive BC-CPH hydrogels [43] (Adapted with permission from Zhou et al. Copyright 2023 Springer Nature). (**C**) Mechanical strength of the conductive BC-CPH hydrogels [43] (Adapted with permission from Zhou et al. Copyright 2023 Springer Nature). (**D**) Demonstration and applications of the conductive BC-CPH hydrogels, exhibiting excellent flexibility and allowing implantation in mice [43] (Adapted with permission from Zhou et al. Copyright 2023 Springer Nature).

Beyond wearable devices, research on conductive hydrogels is steadily expanding toward implantable applications [13]. Hydrogels are soft, elastic, and mechanically compatible with biological tissues, allowing them to conform intimately to organ surfaces after implantation and reducing inflammation caused by mechanical mismatch. Their high water content creates a microenvironment similar to the natural extracellular matrix, enabling excellent biocompatibility and supporting cell adhesion and tissue integration. These characteristics make hydrogels an ideal platform for constructing flexible implantable bioelectronic devices. When conductive components are incorporated, hydrogels evolve from passive structural matrices into active signal interfaces. The embedded conductive network is capable of converting subtle electrical or mechanical signal variations generated externally or within tissues into detectable electrical outputs, thereby enabling conductive hydrogels to function as multimodal biosignal sensors inside the body (Figure 4A) [42]. In addition, conductive hydrogels exhibit favorable interfacial stability and efficient signal transmission in implantable environments, endowing them with broad potential in applications such as neural signal monitoring, cardiac electrophysiological recording, tissue strain sensing, and bioelectrical stimulation. By rationally designing the type and distribution of conductive components and their interactions with the hydrogel network, the electrical properties, mechanical behavior, and biocompatibility of the materials can be further tailored to meet the functional requirements of diverse implantable bioelectronic devices. Nevertheless, for long-term in vivo applications, conductive hydrogels still face challenges including degradation of electrical performance, comprehensive biocompatibility and biosafety evaluation, and long-term interfacial stability, which require systematic investigation in future studies.

In this research area, the series of works by Zhao et al. is particularly influential. Their strategy focuses on fabricating hydrogel-based electronic interfaces with ultrathin geometries while embedding conductive pathways within a continuous hydrogel matrix through printing. Reducing the hydrogel thickness greatly enhances flexibility and conformability to irregular tissue surfaces, thereby minimizing mechanical mismatch after implantation. Encapsulating the conductive components inside the hydrogel also prevents direct exposure to tissues, improving biosafety and electrical stability. Additionally, printing enables precise control over circuit geometry, supporting high-resolution patterning and reliable long-term implantation. For example, Zhao et al. developed a representative system based on a conductive bioink termed BC-CPH, which is formed through phase separation, with PEDOT:PSS serving as the conductive network and hydrophilic polyurethane as the structural matrix (Figure 4B) [43]. This material combines high electrical conductivity with excellent mechanical resilience (Figure 4C) [43]. Through extrusion-based printing, BC-CPH was patterned into flexible circuits and fully encapsulated within a hydrogel matrix, creating hydrogel-only bioelectronic interfaces. With the addition of a rapidly adhering bioadhesive layer, these devices could integrate with tissues without suturing, achieving strong conformal contact while reducing immune responses (Figure 4D) [43]. These devices could be implanted in mice for extended periods without causing noticeable inflammation or adverse tissue responses. This strategy highlights an important direction for the development of implantable hydrogel bioelectronic systems.

## 5. Wound Dressing

**Figure 5:**
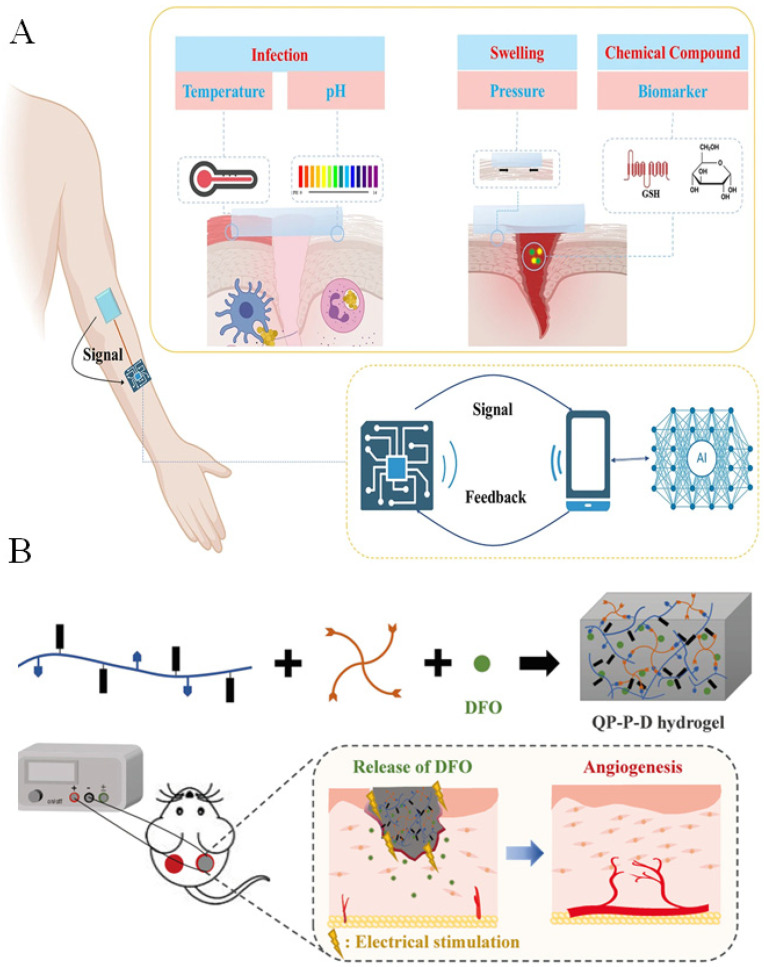
(**A**) Application of conductive hydrogels in wound healing monitoring [46] (Copyright © 2025, She et al. under the terms of the Creative Commons CC BY 4.0). (**B**) Application of conductive hydrogels in accelerating wound repair [48] (Adapted with permission from Wu et al. Copyright 2022 Elsevier).

Conductive hydrogels have also been increasingly applied as wound dressings. Their functions mainly fall into two categories: real-time monitoring of wound status and active promotion of tissue repair [44,45]. During wound healing, key physiological parameters such as temperature, pH, pressure, moisture, and specific biomarkers undergo dynamic changes. Conductive hydrogels can efficiently convert these variations into measurable electrical signals because alterations in the local microenvironment affect ion mobility, network deformation, or the conductive pathways within the hydrogel (Figure 5A) [46]. For example, Wang et al. developed an ionic-liquid-based conductive hydrogel sensor array capable of detecting three-dimensional strain or pressure. Changes in the pressure applied to the wound surface were reflected as shifts in output resistance, enabling real-time monitoring of the healing process when the array was attached to the skin [47].

Conductive hydrogels can also actively accelerate tissue regeneration. Electrical stimulation plays a key role in this process. Endogenous electric fields are known to guide cell migration, enhance fibroblast activity, upregulate angiogenic signaling pathways, and promote epithelialization. When conductive hydrogels deliver mild external electrical cues, they reinforce these natural bioelectrical signals, thereby stimulating cell proliferation, collagen deposition, and neovascularization. For instance, Wu et al. designed a conductive and intrinsically antibacterial hydrogel with pH responsiveness and controlled release of the pro-angiogenic drug deferoxamine (Figure 5B) [48]. The hydrogel enhanced endothelial cell migration and vascularization by upregulating HIF-1α and VEGF. When combined with electrical stimulation, the dressing significantly improved angiogenesis and accelerated the healing of infected diabetic wounds, demonstrating strong therapeutic potential. In addition, conductive hydrogels can modulate reactive oxygen species (ROS) levels at the wound site through their intrinsic electrical conductivity and interaction with endogenous bioelectric fields. By facilitating localized electron transfer and enhancing ionic flux, these hydrogels can influence cellular redox signaling, promoting controlled ROS generation. Moderate ROS levels act as signaling molecules to stimulate angiogenesis, fibroblast proliferation, and collagen deposition, thereby accelerating tissue repair. At the same time, the hydrogel matrix buffers excessive ROS accumulation, reducing oxidative stress and preventing further tissue damage. These combined effects contribute to the accelerated and regulated wound healing process under the influence of conductive hydrogel-mediated bioelectrical cues [49]. These advancements indicate that conductive hydrogels are becoming powerful platforms for intelligent wound monitoring and bioelectrically enhanced tissue regeneration.

## 6. Neural Repair

Conductive hydrogels, with their unique combination of softness, injectability, and electrical functionality, have become promising candidates for nerve tissue engineering among regenerative biomaterials. Neural tissues exhibit pronounced electrophysiological activity, and their development, signaling, and regeneration are all closely governed by electrical cues. Conductive hydrogels, which mimic the structure of the extracellular matrix while simultaneously providing pathways for electrical transmission, therefore create an ideal microenvironment for nerve repair.

**Figure 6:**
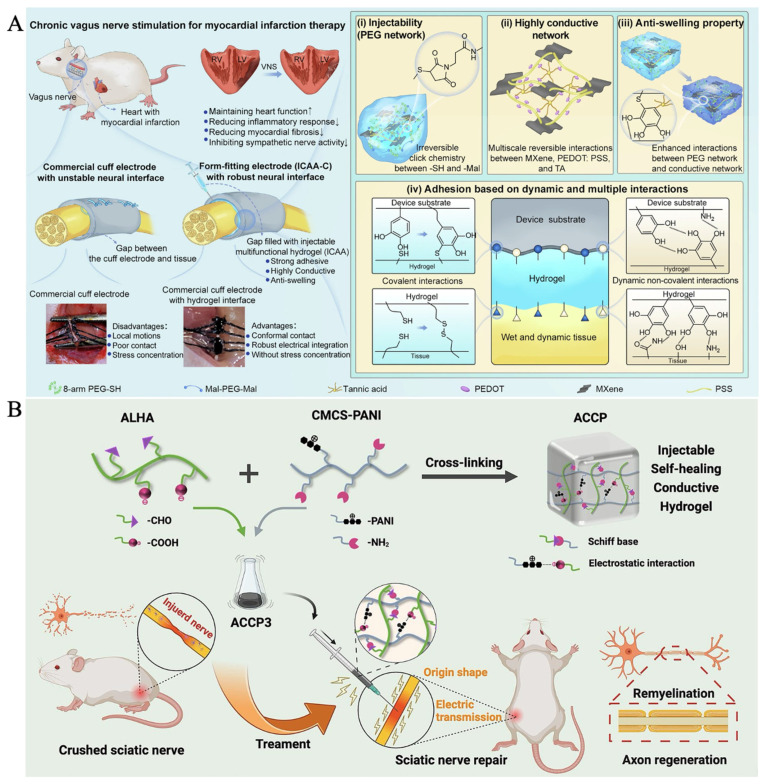
(**A**) Schematic illustration of the form-fitting cuff electrode (ICAA-C) enabled by the ICAA hydrogel for chronic vagus nerve stimulation [50] (Copyright © 2024, Yang et al. under the terms of the Creative Commons CC BY 4.0). (**B**) Design and application of conductive ACCP3 hydrogels for nerve repair [51] (Adapted with permission from Yi et al. Copyright 2023 Elsevier).

The hydrogel matrix provides high water content, tunable mechanical compliance, and excellent tissue compatibility, allowing it to reproduce the soft and hydrated nature of native neural tissue and reduce secondary mechanical injury. Its porous three-dimensional network supports cell adhesion, migration, and axonal extension. The incorporation of conductive components enables the hydrogel to match the electrical conductivity of neural tissues, guiding neuronal firing, promoting Schwann cell proliferation and migration, and enhancing axonal outgrowth. Such an electrically active environment can also regulate ion-channel behavior, modulate growth-factor expression, and support remyelination, thereby accelerating functional recovery. In practical applications, Yang et al. developed a form-fitting, injectable, conductive, adhesive, anti-swelling neural interface (ICAA-C) that can be injected to fill the gap between large commercial cuff electrodes and fine nerves, ensuring conformal contact and robust electrical integration without stress concentration (Figure 6A) [50]. Compared with smaller cuff electrodes that rely on mechanical sutures, the ICAA-C system enables long-term, reliable, and efficient stimulation of fine rat vagus nerves for myocardial infarction treatment. The hydrogel consists of a multifunctional PEG network and a multiscale conductive network. Tannic acid is introduced as a molecular regulator to control click-reaction kinetics, enhance interactions within the conductive network composed of MXene sheets and PEDOT:PSS, and reinforce the overall anti-swelling, electrical durability, mechanical stability, and adhesion to both tissues and device substrates. In another study, Yi et al. synthesized a water-soluble conductive material by grafting polyaniline (PANI) onto carboxymethyl chitosan (CMCS), and prepared dual cross-linked injectable, self-healing, and conductive hydrogels by adding CMCS-PANI (CP) to the dynamic gel network formed by aldehyde-based hyaluronic acid (ALHA) and CMCS through Schiff base reactions, resulting in ALHA/CMCS/CP (ACCP) hydrogels (Figure 6B) [51]. The self-healing ACCP3 hydrogel (3 wt% CP) exhibited good biocompatibility, elastic modulus, and electrical conductivity matching the sciatic nerve. In vitro experiments indicated that ACCP3 promoted the proliferation and migration of Schwann cells. Injection into a sciatic nerve crush injury site reduced tissue resistivity, increased nerve conduction velocity, improved the sciatic nerve functional index, enhanced the expression of neuronal axon-specific proteins, induced axon extension and remyelination, and prevented muscle denervation atrophy. This injectable, self-healing, conductive hydrogel serves as a safe and effective neural tissue substitute, opening new avenues for peripheral nerve injury treatment with potential clinical applications.

## 7. Cell Regulation

As discussed above, conductive hydrogels enhance both wound repair and nerve regeneration, and these advantages largely arise from the favorable effects of electrical cues on cellular behaviors, including proliferation, differentiation, migration, and cytoskeletal organization [52]. Electrical stimulation can modulate membrane potential, activate voltage-gated ion channels, regulate intracellular calcium dynamics, and trigger downstream signaling pathways such as MAPK, PI3K/Akt, and CaMK, thereby influencing how cells grow, communicate, and mature within the hydrogel matrix.

**Figure 7:**
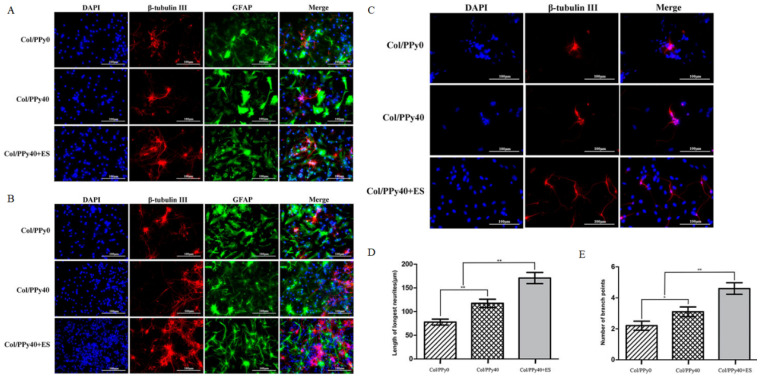
(**A**) Confocal microscopy images of NSCs differentiation on day 3 with the prepared hydrogels [53] (Copyright © 2022, Xu et al. under the terms of the Creative Commons CC BY 4.0). (**B**) Confocal microscopy images of NSCs differentiation on day 7 with the prepared hydrogels [53] (Copyright © 2022, Xu et al. under the terms of the Creative Commons CC BY 4.0). (**C**) Representative fluorescent images of NSC-derived neurons cultured in Col/PPy0, Col/PPy40, and Col/PPy40 + ES (200 mV) groups [53] (Copyright © 2022, Xu et al. under the terms of the Creative Commons CC BY 4.0). (**D**) Neurite length of NSC-derived neurons in different groups. Data are presented as mean ± SD. * *p* < 0.05, ** *p* < 0.01 [53] (Copyright © 2022, Xu et al. under the terms of the Creative Commons CC BY 4.0). (**E**) Number of branch points of NSC-derived neurons in different groups [53] (Copyright © 2022, Xu et al. under the terms of the Creative Commons CC BY 4.0). All scar bars have a length of 100 μm.

Building on these principles, Xu et al. investigated how conductive hydrogels regulate neural stem cell (NSC) behavior by comparing a non-conductive collagen hydrogel (Col/PPy0), a conductive collagen–polypyrrole hydrogel (Col/PPy40), and the conductive hydrogel combined with electrical stimulation (Col/PPy40 + ES). At both day 3 and day 7, NSCs cultured on Col/PPy40 with or without ES showed markedly increased expression of the neuronal marker β-tubulin III and reduced expression of the astrocyte marker GFAP compared with Col/PPy0, indicating enhanced neuronal differentiation. The strongest neuronal differentiation occurred in the Col/PPy40 + ES group (Figure 7A,B) [53]. Beyond lineage specification, electrical stimulation also promoted neuronal maturation (Figure 7C) [53], as neurons derived from NSCs exhibited significantly longer neurites and more branch points in the Col/PPy40 + ES group compared with either Col/PPy40 or Col/PPy0, demonstrating that conductive hydrogels combined with electrical cues synergistically enhance neurite extension and network formation (Figure 7D,E) [53]. These findings highlight the important role of conductive hydrogels as microenvironments capable of directing cell–cell interactions and promoting electrically responsive cell functions.

## 8. Discussion and Outlook

Although conductive hydrogels have achieved significant advances in biomedical applications such as wearable electronics, implantable biointerfaces, wound healing, neural regeneration, and cell regulation, their further development requires more sophisticated material design strategies and precise engineering approaches. One of the core challenges is how to maintain electrical conductivity while simultaneously meeting multiple critical performance requirements. For wearable devices, hydrogels must combine strong and reversible adhesion, high extensibility and toughness, long-term biocompatibility, inherent antibacterial properties, and freeze resistance at low temperatures to ensure stable operation in complex daily environments. For in vivo applications, higher requirements include intimate tissue integration, excellent biocompatibility, low immunogenicity, appropriate degradability, and long-term electrical stability in physiological conditions. Achieving a balance among these properties is challenging because enhancing one property often compromises another. In addition, safety challenges need to be carefully considered, including potential nanoparticle toxicity, inflammatory responses, and the effects of biodegradation products released during hydrogel degradation. These factors can influence both short-term biocompatibility and long-term tissue responses, potentially affecting the performance and reliability of the implanted device. Moreover, sterilization compatibility is critical for clinical translation. Common sterilization methods, such as gamma irradiation, autoclaving, and ethanol treatment, must be carefully evaluated, as each technique can impact the hydrogel’s mechanical properties, electrical conductivity, and structural integrity. Ensuring that hydrogels retain their functional performance after sterilization while avoiding the introduction of cytotoxic residues or altered degradation profiles is essential for safe and effective clinical applications. Together, these considerations highlight the importance of rigorous preclinical testing and material optimization to address both biosafety and regulatory requirements for translational use.

At the fabrication level, traditional approaches that rely solely on simple doping of conductive components are no longer sufficient to achieve precise microstructural control and complex functional integration. Advanced manufacturing strategies, such as three-dimensional printing, micro-extrusion patterning, and photolithography-based structuring, will play a crucial role in the future. These methods enable precise spatial organization of conductive networks, construction of anisotropic or gradient architectures, and highly integrated sensing, stimulation, and tissue interface functions, significantly enhancing the reliability and functional complexity of conductive hydrogels. The multimodal sensing capability of conductive hydrogels represents another key direction for future development. Many current hydrogel systems still primarily rely on electromechanical responses induced by stress, while their ability to simultaneously detect temperature, humidity, chemical signals, or biomolecules remains limited. By incorporating multifunctional material components or designing gradient and partitioned structures, conductive hydrogels can achieve multimodal detection of electrical, mechanical, chemical, and biological signals within a single system. This capability not only enhances the performance of wearable and implantable devices but also provides richer feedback for tissue repair and cell regulation, enabling the construction of more precise and controllable bioelectronic systems.

Looking ahead, the further development of conductive hydrogels will rely on multifunctional design and deeper mechanistic understanding. Beyond conductivity, hydrogels can integrate dynamic biochemical signaling modules, mechanoresponsive units, immunomodulatory functions, or drug delivery components to enable simultaneous sensing, response, and promotion of tissue repair. A more comprehensive understanding of the synergistic interactions among electrical, mechanical, and chemical cues at the cell–material interface will facilitate rational material design. Therefore, the deep integration of materials science, biomedical engineering, and advanced manufacturing technologies will be critical for translating conductive hydrogels from conceptual research into clinical and industrial applications.

## 9. Conclusions

Conductive hydrogels show significant potential in biomedical applications, owing to their combination of tissue-like softness, high water content, biocompatibility, and electrical conductivity. By incorporating ionic or electronic conductive pathways, these hydrogels can transmit and modulate electrical signals while supporting dynamic interactions with biological tissues, enabling applications in wearable electronics, implantable biointerfaces, wound healing, neural regeneration, and cell regulation. Despite these advances, challenges remain in achieving a balance among mechanical robustness, adhesion, long-term stability, biocompatibility, and multifunctionality. Traditional fabrication methods relying solely on simple doping of conductive components are often insufficient for constructing complex architectures or integrating multiple functions. Advanced manufacturing strategies, including three-dimensional printing, micro-patterning, and photolithography, provide precise control over microstructure, conductive networks, and spatial organization, enhancing both functionality and reliability. Future development of conductive hydrogels will likely focus on multifunctional design and multimodal sensing, incorporating biochemical signaling, mechanoresponsive elements, immunomodulatory functions, and controlled drug delivery. A deeper mechanistic understanding of the synergistic interactions between electrical, mechanical, and chemical cues at the cell–material interface will facilitate rational design and optimization. Through interdisciplinary efforts in materials science, biomedical engineering, and advanced manufacturing, conductive hydrogels are expected to transition from laboratory research to practical clinical and industrial applications, offering transformative solutions for next-generation conductive hydrogel systems.

## Figures and Tables

**Table 1:** Comparison of ionic, electronic, and mixed conductive hydrogels [33,35].

Conduction Type	Charge Carriers	Typical Components	Typical Conductivity Range (S·cm^−1^)
Ionic	Mobile ions (e.g., Na^+^, K^+^, Cl^−^)	Salt-doped hydrogels, polyelectrolytes, zwitterionic polymers	~10^−1^–10^3^
Electronic	Electrons	Conductive polymers (e.g., PEDOT:PSS), graphene, carbon nanotubes, metal fillers	~10^−2^–10^2^
Mixed (ionic–electronic)	Ions + electrons	Hybrid hydrogels combining ionic networks with conductive polymers or fillers	~10^−2^–10^3^

## Data Availability

Not applicable. As this article is a review article, the data supporting this article can be found in the original articles discussing each topic.
